# Is Right Unilateral Transversus Abdominis Plane (TAP) Block Successful in Postoperative Analgesia in Laparoscopic Cholecystectomy?

**DOI:** 10.1155/2022/2668215

**Published:** 2022-04-06

**Authors:** Serhat Ozciftci, Yeliz Sahiner, Ibrahim Tayfun Sahiner, Taylan Akkaya

**Affiliations:** ^1^Hitit University Faculty of Medicine, Department of Anesthesiology and Reanimation, Çorum Merkez, Turkey; ^2^Hitit University Faculty of Medicine, Department of General Surgery, Çorum Merkez, Turkey; ^3^University of Health Science, Dışkapı Yıldırım Beyazıt Training and Research Hospital, Department of Anesthesiology and Reanimation and Pain Clinic, Ankara, Turkey

## Abstract

**Background:**

Transversus abdominis plane (TAP) block is used for postoperative analgesia in laparoscopic cholecystectomy. In laparoscopic cholecystectomy, the incisions are located mainly on the upper right side of the abdomen.

**Aims:**

We aim to determine the efficacy of less-invasive ultrasound-guided right unilateral oblique subcostal TAP block in laparoscopic cholecystectomy on postoperative analgesia by comparing patients undergoing bilateral TAP block and a control group.

**Methods:**

Ninety patients were equally divided into control, unilateral, and bilateral TAP block groups. TAP blocks were conducted before anesthesia. No block was applied to the control group. Patients' demographics and postoperative pain, satisfaction, and nausea-vomiting scores and tramadol/ondansetron doses were evaluated.

**Results:**

There was no significant difference in the verbal numerical rating scale for pain scores at rest and during coughing (VNRS-R and VNRS-C) between unilateral and bilateral TAP block groups at postoperative 1 hour, 2 hour, 4 hour, 8 hour, 12 hour, and 24 hours. In addition, VNRS-R and VNRS-C scores were significantly higher in the control group than in the other two groups. Tramadol consumption in the control group was significantly higher than in the unilateral and bilateral TAP block groups (*p* ≤ 0.01), while no significant difference was identified between unilateral and bilateral TAP block groups (*p*=0.303). Nausea-vomiting scores and ondansetron consumption did not differ significantly between all the groups. Patient satisfaction was significantly higher in unilateral and bilateral groups (*p* < 0.01, *p* < 0.01) than in the control group, while there was no significant difference between unilateral and bilateral TAP block groups (*p*=0.793).

**Conclusions:**

Right unilateral TAP block provides postoperative analgesia as effective as bilateral TAP block in laparoscopic cholecystectomy.

## 1. Introduction

Laparoscopic cholecystectomy (LC) is a commonly performed, minimally invasive surgical approach. Abdominal pain occurring after laparoscopic surgery can be associated with port-site incision, diaphragmatic irritation resulting from carbon dioxide (CO_2_) insufflation, and incomplete evacuation of insufflated gas. Pain occurring after laparoscopic abdominal surgery can take three forms: incisional pain (somatic pain), visceral pain (deep intra-abdominal pain), and shoulder pain (reflected visceral pain) [1]. Among these components, it is the somatic pain pathways that are inhibited by TAP blocks [2, 3]. The somatic pain in the abdominal wall is transmitted through the ipsilateral thoracolumbar fibers [4]. The four incisions in the LC are located predominantly on the right side. Therefore, incisional pain in LC originates predominantly from the right side.

The transversus abdominis plane (TAP) block is a regional anesthesia method used in abdominal surgery for postoperative analgesia [5]. The TAP block reduces postoperative narcotic consumption and nausea-vomiting, improves respiratory functions, increases patient satisfaction, and contributes to early mobilization and discharge of patients [6–10]. Oblique subcostal TAP block provides more effective analgesia than other TAP block methods in LC [11].

The aim of this study is to compare the effects of right-sided unilateral TAP block on postoperative analgesia, nausea-vomiting, and patient satisfaction with the bilateral TAP block group and control group. Thus, complications can be decreased by reducing the number of injections and the amount of local anesthetic consumed with unilateral TAP block.

## 2. Materials and Methods

This prospective, randomized, and single blind clinical trial was conducted in the Hitit University Erol Olcok Training and Research Hospital, Corum, Turkey, between September 01, 2017, and June 01, 2019, after obtaining written consent of the patients and approval of the Hitit University Faculty of Medicine Local Ethics Committee (August 15, 2017–66). Regional anesthesia and general anesthesia were administered by different teams, LC was performed by a single senior surgeon, and pain/other score assessment was performed by ward nurses. The general anesthesia team, ward nurses, physicians, and surgeon were blind to study groups.

### 2.1. Patient Selection and Groups

**Figure 1:**
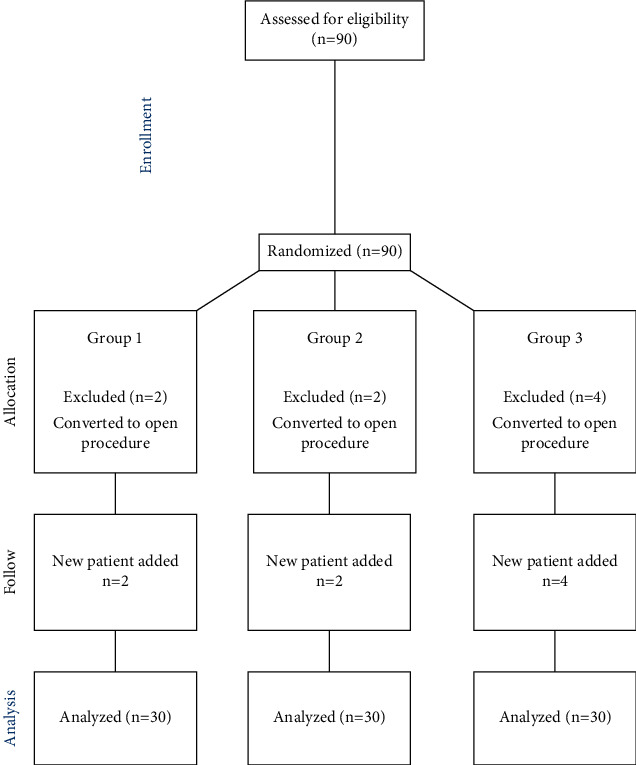
Flowchart of the study.

The randomization scheme was generated by using the website Randomizer (https://www.randomizer.org). As dictated by the study power and calculated using statistical methods, a total of 90 patients were equally randomized into three groups. Patients who did not undergo a TAP block were assigned to the control group (Group 1, *n* = 30); those undergoing a right-sided unilateral TAP block were defined as Group 2 (*n* = 30); and those undergoing a bilateral TAP block were defined as Group 3 (*n* = 30). But, two patients in Group 1, two patients in Group 2, and four patients in Group 3 were excluded due to the conversion of these patients to open cholecystectomy, and additional eight patients were recruited to maintain a constant sample size of 30 patients in each group (Figure 1).

Included in the study were 90 patients aged 18–80 years with an American Society of Anesthesiologists (ASA) status of I, II, or III and who were scheduled for elective LC.

Patients who were unable to rate their pain on a verbal numerical rating scale (VNRS), those with a history of allergy to local anesthetics and administered drugs, those with presence of an infection or inflammation at the proposed site of injection, those with liver or kidney failure, alcohol, opioid, or substance addiction, those with chronic pain disorders and the chronic use of analgesics, those with severe systemic disease, those with a coagulation disorder, those with body mass index (BMI) ≥ 35, those with lack of consent to participate in the study, patients who were pregnant and breastfeeding, and those with a history of abdominal surgery or emergency surgery were all excluded from the study.

Patients who were converted from laparoscopic surgery to open surgery, patients who withdrew their consent at any stage of the study, and patients with a VNRS score of 7 or higher four hours after surgery when other opioids will be used for analgesia were excluded from the study.

Pain (at rest and with cough and shoulder pain), side effects associated with the use of narcotic analgesics, such as nausea-vomiting, the amount of tramadol and ondansetron consumed, and patient satisfaction were all evaluated after surgery.

The VNRS was used to evaluate pain level, for which all patients were informed of the pain scale in detail in the preoperative period (0 = no pain at all, 10 = the worst pain that one can ever imagine).

The time between the procedure and the start of surgery was recorded as the Block Time. The postoperative nausea and vomiting (PONV) scale was used to evaluate nausea and vomiting after surgery (1: no nausea, no vomiting; 2: nausea present, no vomiting; 3: nausea present, vomiting once; 4: nausea present, vomiting twice or more or continuous retching), and a 5-point Likert-type scale was used to evaluate patient satisfaction. Shoulder pain was rated as “present” or “absent.” These parameters were evaluated at 1 hour, 2 hours, 4 hours, 8 hours, 12 hours, and 24 hours after surgery. Patient satisfaction was recorded 24 hours after surgery. All parameters was carried out by the service nurses.

Paracetamol 1 g was administered as an intravenous infusion at 6 hour, 12 hour, 18 hour, and 24 hour after surgery, and all patients were administered intramuscular diclofenac sodium at 12 hour and 24 hour after surgery as routine to provide analgesia. A tramadol infusion at a dose of 0.5 mg/kg was administered once per hour if the patient reported a VNRS-R score of 4 or higher at rest. The maximum tramadol dose was 500 mg/day. Patients with a PONV score of 3 points and higher were administered 4 mg of ondansetron at 4-hour intervals. The total amounts of tramadol and ondansetron consumed after surgery were recorded.

### 2.2. Administration of TAP Block

The patients in the three groups underwent standard monitoring (pulse oximetry, electrocardiogram, and noninvasive blood pressure) in the preoperative room. A venous line was installed using a 20-gauge catheter, and a 500 ml physiological serum infusion was initiated. All patients underwent premedication with intravenous midazolam at a dose of 0.02 mg/kg, after which the patients in the control group were transferred directly to the operating room. The regional anesthesia team performed all TAP block procedures. The TAP block was applied in the preanesthesia period for preemptive analgesia. Decreased sensation in the appropriate dermatomal levels in TAP block groups was confirmed by pinprick before general anesthesia by the regional anesthesia team. The TAP blocks were performed with a GE LOGİQ V2, GE Medical Systems, Jiangsu, China, ultrasound system and linear ultrasound transducer (6–12 Hz). For the TAP block procedure, the rectus abdominis muscle and fascia were detected 2 cm inferior to the xiphoid using a linear probe. The linear probe was moved inferior-laterally from the xiphoid to the superior anterior iliac crest over the oblique subcostal angle, and the external oblique, internal oblique, and transversus abdominis muscles and fascia were located. A 1-2 ml of 2% lidocaine was then applied to the insertion site of the peripheral block needle. While the probe was in the oblique subcostal position, the peripheral block needle (Braun, Ultra360, 100 mm, Germany) was advanced from the medial to the inferolateral area of the probe along the fascia of the transversus abdominis muscle and the internal oblique muscle, and the block area was confirmed through the infiltration of 1-2 ml of a physiological serum solution to create a hypoechoic and biconvex appearance, as mentioned in [11]. Patients in the unilateral block group received 20 ml of 0.25% bupivacaine on the right side of the abdomen as given in Sahin et al. [12], whereas the patients in the bilateral block group received a total of 40 (20 + 20) ml of 0.25% bupivacaine at both sides of the abdomen. Performing unilateral TAP block would both save time and could reduce consumption of local anesthetic. All patients were transferred to the operating room after the completion of the block procedure.

### 2.3. General Anesthesia Procedure

Patients underwent standard monitoring in the operating room. General anesthesia was administered by the general anesthesia team. The general anesthesia protocol with sevoflurane was applied to all patients. No additional ketorolac, long-acting opioids, ketamine, or local anesthetic infusions were administered during the surgical procedure. The patients received an intravenous (IV) infusion of paracetamol 1 g, IV tramadol 2 mg/kg, intramuscular diclofenac sodium 75 mg, and an IV infusion of ondansetron 4 mg during surgery to relieve postoperative pain, nausea, and vomiting. The patients were extubated after surgery and moved to the recovery unit. Then patients were transferred to the ward.

### 2.4. Surgical Procedure

The same senior surgeon who was blind to the groups of the patients performed all operations. A 10 mm incision was made in the midline, parallel to the pelvis, 2 cm below the xiphoid; a 10 mm periumbilical incision was made parallel to the pelvis; and two 5 mm subcostal incisions were made for trocar insertion during laparoscopic cholecystectomy. The surgery was carried out laparoscopically, with CO_2_ insufflation pressure limited to 10–12 mmHg.

### 2.5. Statistical Methods

The necessary sample size in the groups was calculated through a power analysis before starting the study (power = 0.80). Power Analysis and Sample Size Software (PASS) was used in the power analysis (version 11 trial version). Taking advantage of the studies in the literature for the calculations of the estimated sample size, the sample average for the three groups was 6, 5.5, and 5 with a standard deviation of 1.5 for the inclusion of an equal number of patients in each group. The alpha was set to 0.05 (95% level of significance). The power analysis determined a total of 90 patients to be included, with 30 patients in each group, meaning an effect size of 0.373 and a power of 81% (0.81).

In the present study, the statistical analysis was performed using the SPSS (Version 22.0, SPSS Inc., Chicago, IL, USA, licensed to Hitit University) software package. Descriptive statistics included mean ± standard deviation for normally distributed continuous variables, median (min-max) for abnormal distributions and ordinal variables, and number and percentage for categorical variables. A Kolmogorov–Smirnov test and a Shapiro–Wilk test were used to evaluate the normality of distribution. Levene's test was used to examine the homogeneity of variances. An independent samples *t*-test was used to compare the mean values of the independent samples for continuous variables, and a Mann–Whitney *U* test was used to analyze variables without a normal distribution. Nominal variables were analyzed using a Chi-square test. One-way analysis of variance (ANOVA) was used for the comparison of more than two groups, and a Kruskal–Wallis test was used to analyze ordinal variables (pain scores). After performing a Kruskal–Wallis test, a Mann–Whitney *U* test was used with a Bonferroni correction to determine the group that caused the most significant difference (post hoc). A *P* value of less than 0.05 was considered statistically significant.

## 3. Results

In this study, morbidity due to interventional procedures did not occur. A total of 90 patients were evaluated, with 30 cases in each group.

All groups were compared for age, gender, ASA, BMI, duration of anesthesia, and duration of surgery. We found no statistically significant difference among the groups in terms of age, gender, body mass index, and anesthesia and surgery duration. Furthermore, the Block Time from the administration of the TAP block to the onset of surgery was evaluated. No significant difference was found in the Block Time duration of Group 2 and Group 3 (Table 1).

When the pain scores of all groups were evaluated at rest and during coughing (VNRS-R and VNRS-C), significant differences were found between the groups at 1 hour, 2 hour, 4 hour, 8 hour, 12 hour, and 24 hours (Table 2). The groups were compared in a paired fashion. The VNRS-R and VNRS-C scores were compared between the unilateral and bilateral block groups and the control group at all time points, and it was found that the VNRS-R and VNRS-C scores were significantly higher in the control group than in the other two groups (Graphic 1, 2). The VNRS-R and VNRS-C scores did not differ significantly between the unilateral and bilateral block groups at any of the time points (Table 2). The VNRS-R scores at 1 hour, 2 hour, 4 hour, and 12 hours and the VNRS-C scores at 1 hour were lower in the unilateral block group than in the bilateral block group, but not to a statistically significant degree (Graphic 1, 2). A comparison was made between the three groups in terms of opioid consumption, and a statistically significant difference was found. When the groups were compared in pairs, the total amount of opioids consumed in the control group was significantly higher than in the unilateral and bilateral block groups, while the total amount of opioids consumed did not differ significantly between the unilateral and bilateral block groups (Table 1). The lowest tramadol consumption was observed in the unilateral block group.

There was a significant difference between the groups in terms of radiating shoulder pain only at 24 hours (*p*=0.01). A paired comparison of the groups revealed a significant difference in shoulder pain between the control group and unilateral (*p*=0.006) and bilateral (*p*=0.006) block groups at 24 hours, with shoulder pain being more common in the control group at 24 hours than in the other two groups. The presence of shoulder pain did not significantly differ between the unilateral and bilateral block groups (*p*=1.000).

A statistically significant difference was observed in patient satisfaction between all three groups (*p*=0.001), for which a paired comparison was made. Patient satisfaction was higher in the unilateral and bilateral block (*p* < 0.01, *p* < 0.01) groups than in the control group, and the difference was significant. There was no significant difference in patient satisfaction between the unilateral and bilateral block groups (*p*=0.793) although patient satisfaction was remarkably higher in the unilateral block group.

There was no significant difference between the groups in PONV scores and ondansetron consumption (respectively, *p* *=* *0.934* and *p* *=* *0.410*), although the amount of ondansetron consumed was lowest in the unilateral block group.

No significant difference was found between the three groups in terms of surgery and anesthesia duration (Table 1).

## 4. Discussion

There are many studies in the literature evaluating the effectiveness of unilateral or bilateral TAP block in various procedures [13]. However, there is no study comparing the effectiveness of right unilateral TAP block on postoperative analgesia, nausea-vomiting, and patient satisfaction in laparoscopic cholecystectomy with that of bilateral TAP block and control groups. In addition, reducing the number of injections and the amount of local anesthetic consumed will decrease complications.

In LC, the incision and surgical procedure are made on the right side of the abdomen. Therefore, right unilateral TAP block can be expected to provide analgesia equal to bilateral TAP block. A control group without a block was included in the study to compare the postoperative efficacy of unilateral and bilateral TAP blocks. We evaluated the effectiveness of unilateral TAP block as well as bilateral TAP block in terms of postoperative pain scores (VRNS-R and VRNS-C), opioid consumption, nausea-vomiting scores, and patient satisfaction, and we did not find a statistically significant difference in all parameters between these two block groups.

Elnabtity et al. evaluated the effectiveness of unilateral TAP block in unilateral ureteric extracorporeal shock wave lithotripsy (ESWL), which causes both superficial and visceral pain, in two different studies [14, 15]. In [14], patients with unilateral TAP block and patients who were not blocked were compared, and in their other study [15], unilateral TAP block and bilateral TAP block were compared. In the first study, it was determined that unilateral TAP block provided a significant decrease in pain scores. In the second study, no significant difference was found between unilateral TAP block and bilateral TAP block in terms of pain scores. These two studies by Elnabtity et al. and our study support that ipsilateral TAP block can provide effective analgesia in surgical and nonsurgical procedures that cause unilateral somatic pain.

Hotujet and others compared the effects of a unilateral TAP block, applied with a single-port entry, with a placebo in patients undergoing robotic gynecological surgery, and reported that the TAP block provided effective analgesia and significantly reduced the amount of opioids used in robotic surgery [16]. The authors thus concluded that a unilateral TAP block in laparoscopic interventions via a unilateral incision provides effective analgesia. In our study, we showed that unilateral TAP block provides effective analgesia not only in unilateral incisions but also in LC where the incisions are predominantly unilateral.

Lee et al. [17] reported that a unilateral TAP block performed under sedation in patients undergoing the open gastrostomy procedure might be effective in the pain and anesthesia management of the patients. In addition, Yamamoto et al. [18] reported that a unilateral TAP block that does not block visceral pain could be used as a method of anesthesia in combination with sedation and local infiltration in patients undergoing a peritoneal dialysis catheter insertion performed through a unilateral incision. Lee et al. and Yamamoto et al. showed that unilateral TAP block provides effective analgesia in open surgical procedures involving one side of the abdomen. We also found that unilateral TAP block provides effective analgesia not only in open surgeries but also in LC, which is one of the laparoscopic interventions.

Right unilateral TAP block in LC reduces resting pain scores and opioid consumption [19, 20]. While Tolcard and others [19] found a decrease in side effects in terms of patient discharge due to less opioid use in the subcostal TAP block group (subcostal TAP block vs port-site infiltration), Arık and others [20] found no difference between the groups (unilateral, port-site infiltration, and control) in terms of nausea and vomiting. However, since these two studies compared unilateral TAP block with port-site infiltration, these studies do not provide information on whether unilateral TAP block is as effective as bilateral TAP block. Our study showed that unilateral TAP block is as effective as bilateral TAP block in postoperative analgesia and unilateral TAP block reduces postoperative opioid consumption and nausea-vomiting as much as bilateral TAP block. In addition, although it was not statistically significant, patient satisfaction was higher in the unilateral block group than in the bilateral block group. We think that the higher patient satisfaction in the unilateral group is due to the less invasiveness of the unilateral TAP block. However, the fact that all blocks were performed on awake patients in our study may have affected this situation.

Laparoscopic cholecystectomy is the gold standard surgical method in gallbladder diseases. Since it is a minimally invasive approach, it provides an advantage in terms of wound complications and especially postoperative pain. Although it is a minimally invasive approach, especially in patients who have undergone laparoscopic upper abdominal surgery, severe shoulder pain may occur in the postoperative period, which negatively affects patient comfort. Although effective removal of CO_2_ at the end of the surgical procedure is an important maneuver in preventing this pain, postoperative analgesic and opioid use is often required [21]. In this study, the differences of the patients in terms of postoperative shoulder pain were evaluated and a statistically significant difference between the groups was found only at 24 hours. Although there is no statistical difference, it has been determined that TAP block applications contribute to patient comfort clinically. Based on this clinical observation, TAP block applied to patients not only contributed to patient comfort in the postoperative period but also prevented unnecessary analgesic and opioid use.

It is known that bilateral TAP block applied using ropivacaine has significant effects on coughing pain scores in the early postoperative period [22]. However, this positive effect was at a low level. In our study, in which we used bupivacaine, there was a statistically significant decrease in VRNS-C scores in both unilateral and bilateral TAP block groups compared to the control group in the first 24 hours. Also, there was no statistically significant difference between unilateral and bilateral TAP block groups.

In the study to determine the optimal local anesthetic volume and concentration in LC, unilateral TAP block was applied in different volumes and concentrations (50 mg 0.5% bupivacaine+10 ml serum physiologic or 50 mg 0.5% bupivacaine+20 ml serum physiologic) and it was determined that TAP block was a part of balanced postoperative analgesia [12]. However, this study did not reveal whether unilateral TAP block was effective alone. We determined that the low-dose and concentrated bupivacaine solution used in that study was effective in postoperative analgesia by comparing it with the control and bilateral TAP groups.

The VNRS scoring system, which is partially relative, was used to evaluate the pain scores of the patients. We could not apply patient-controlled analgesia (PCA) because there were not enough PCA devices in our hospital. The patient-controlled analgesia will further strengthen the study. In addition, performing TAP block under general anesthesia may be better for patient comfort.

It will be very important to investigate the effectiveness of ipsilateral TAP block in other studies in laparoscopic procedures such as laparoscopic herniorrhaphy and laparoscopic appendectomy, which are predominantly performed with unilateral incisions.

## 5. Conclusion

In laparoscopic cholecystectomy, right unilateral oblique subcostal TAP block provides a decrease in postoperative pain scores and tramadol consumption and an increase in patient satisfaction like bilateral TAP block. In conclusion, right unilateral oblique subcostal TAP block, which is less invasive and a local anesthetic consumed less than bilateral TAP block, can be applied for postoperative analgesia in laparoscopic cholecystectomy.

## Figures and Tables

**Table 1:** Demographic data and surgery, anesthesia, and block performing time.

		Group 1 Control (n:30) Mean± standard deviation	Group 2 Unilateral TAP block (n:30) Mean± standard deviation	Group 3 Bilateral TAP block (n:30) Mean± standard deviation	*P* value
Age		47,46±11,83	48,46±12,05	51,90±11,40	0,315^a^
Sex	Male	9	7	8	0.954^b^
	Female	21	23	22	
ASA score	I	8	7	4	0,197^b^
	II	22	19	23	
	III	0	4	3	
Anesthesia time (min)		67,36±17,47	64,83±16,92	68,36±21,99	0,769^c^
Surgery time (min)		50,36±17,03	45,73±17,10	49,53±18,62	0,509^c^
Body mass index		28,95±3,12	29,81±3,48	29,63±3,14	0,505^c^
Total ondansetron (mg)		1,73±2,91	0,93±2,91	1,46±3,05	0,163^c^
Total tramadol (mg)		93,13±61,86	20,66±29,23	27,66±29,90	<0.001^c^
Block Time (time from the administration of TAP block to surgery, min)		-	43,60±18,40	49,83±21,00	0,264^d^

^a^Analysis of variance (ANOVA), ^b^Fisher's Exact Test, ^c^Kruskal–Wallis test, ^d^Mann–Whitney U test, ASA: American Society of Anesthesiologists, TAP: transversus abdominis plane, min: minute, and mg: milligram.

**Table 2:** Postoperative VNRS-R and VNRS-C scores.

Postoperative VNRS		Group 1 Control (n:30) Mean± standard deviation; median (Min-Max)	Group 2 Unilateral TAP block (n:30) Mean± standard deviation; median (Min-Max)	Group 3 Bilateral TAP block (n:30) Mean± standard deviation; median (Min-Max)	*P* value	Post hoc
1^st^ hour	Rest	6,03±1,520	3,10±1,605	3,33±1,061	<0,001^a^	1-2: <0.001
		6(3-9)	3(1-6)	3(2-6)		1−3: <0.001
	Cough	6,33±1,807	3,37±1,771	3,47±1,479	<0,001^a^	1-2: <0.001
		7(3−9)	3(1−8)	3(0−6)		1−3: <0.001

2^nd^ hour	Rest	4,50±1,737	2,70±1,208	2,87±1,224	<0,001^a^	1-2: <0.001
		4(2−8)	2(1−5)	3(1−6)		1−3: 0.001
	Cough	3,33±1,688	5,23±2,063	3,20±1,349	<0,001^a^	1-2: 0.001
		5(2−9)	3(1−7)	3(1−6)		1−3: 0.001

4^th^ hour	Rest	2,80±1,186	1,83±,834	2,17±0,791	0,001^a^	1-2: 0.001
		3(1−6)	2(1−4)	2(1−4)		
	Cough	3,63±1,586	2,73±1,337	2,53±1,008	0,014	1-2: 0.022
		3(1−7)	2(1−6)	3(1−5)		

8^th^ hour	Rest	2,60±1,133	1,83±0,834	1,73±0,740	0,002^a^	1-2: 0.004
		2,50(1−6)	2(1−3)	2(1-3)		1−3: 0.017
	Cough	3,57±1,455	2,57±1,278	2±0,743	<0,001^a^	1-2: <0.001
		3,50(1−6)	2(1−6)	2(1−3)		
						1−3: 0.021
12^th^ hour	Rest	2,17±1,020	1,40±0,770	1,63±0,890	0,009^a^	1-2: 0.007
		2(1−5)	1(0-3)	1,50(0-3)		
	Cough	3,13±1,525	2±1,203	1,83±1,020	0,001^a^	1-2: 0.002
		3(1−6)	2(0-5)	2(0-4)		
						1−3: 0.010

24^th^ hour	Rest	2,10±1,094	1,23±0,679	1,23±0,898	<0,001^a^	1-2: 0.001
		2(1−6)	1(0−3)	1(0−3)		1−3: 0.002
	Cough	3,07±1,701	1,77±1,006	1,57±1,135	<0,001^a^	1-2: <0.001
		3(1−8)	1,50(0−4)	1(0−5)		1−3: 0.004

^a^Kruskal–Wallis test, VNRS: verbal numerical rating scale (rest and cough (VNRS-R and VNRS-C)), Min: minimum, Max: maximum, and TAP: transversus abdominis plane.

## Data Availability

No data were used to support this study.

## Supplementary Materials

Supplementary Materials Graphic 1: verbal numeric rate scale (VNRS) rest score. Graphic 2: verbal numeric rate scale (VNRS) cough score.
