# Supplementary figures and images for: Is Right Unilateral Transversus Abdominis Plane (TAP) Block Successful in Postoperative Analgesia in Laparoscopic Cholecystectomy?
Source: Int J Clin Pract. 2022 Apr 6;2022:2668215. doi: 10.1155/2022/2668215 (PMC9159215; doi:10.1155/2022/2668215)

VNRS Rest Score

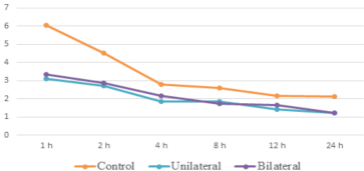

Supplement: Supplementary Materials — Graphic 1: verbal numeric rate scale (VNRS) rest score. Graphic 2: verbal numeric rate scale (VNRS) cough score. [file 2668215.f1.zip › Graphic 1.pdf]

VNRS Cough Score

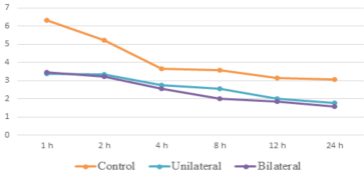

Supplement: Supplementary Materials — Graphic 1: verbal numeric rate scale (VNRS) rest score. Graphic 2: verbal numeric rate scale (VNRS) cough score. [file 2668215.f1.zip › Graphic 2.pdf]
